# Solitary fibrous tumour of the liver—report on metastasis and local recurrence of a malignant case and review of literature

**DOI:** 10.1186/s12957-017-1102-y

**Published:** 2017-01-18

**Authors:** Nelson Chen, Kellee Slater

**Affiliations:** Department of Hepatobiliary and Pancreatic Surgery, Princess Alexandra Hospital, 199 Ipswich Road, Woolloongabba, QLD 4102 Australia

**Keywords:** Solitary fibrous tumour of the liver, Hepatic tumours, Mesenchymal neoplasms, Malignancy, Metastasis, Hepatobiliary

## Abstract

**Background:**

Solitary fibrous tumours (SFT) are neoplasms of mesenchymal origin that predominantly arise from the pleura. SFT of the liver (SFTL) are a rare occurrence with little number of cases reported in English literature. Malignant cases of hepatic SFT are an even rarer occurrence. For this reason, the prognostic evaluation of SFTLs is unknown and difficult to measure.

**Methods:**

A search on English literature on “Solitary Fibrous Tumour of the Liver” was conducted on common search engines (PubMed, Google). All published articles, case reports and literature reviews and their reference lists were reviewed.

**Case report:**

This paper presents a 61-year-old male who was referred to a tertiary hospital in April 2010 with marked hepatomegaly. USS, CT and MRI scans were suggestive of a neoplasm, and the patient underwent a subsegmental IVb resection in June 2010. The specimen demonstrated histological and immunohistochemical features of malignant SFTL with clear resection margins. The patient was followed up regularly for 3 years with imaging and no suggestion of recurrence. Six years after the initial surgery, the patient represented with worsening right upper quadrant pain and dyspnoea secondary to extensive tumour recurrence adjacent to the resection site and metastatic deposits in the pleura. The patient was managed symptomatically and discharged for community follow-up after palliative involvement.

**Conclusions:**

SFTL are rare with only 84 cases reported in the English Literature including the present case. The average age of patients is 57.1 and occurs in females more than males (1.4:1). Most SFTLs follow a benign course, however, 17.9% of cases displayed malignant histological features. Only three cases including the current case are reported to have both local recurrence and metastasis. Surgical resection remains the mainstay of treatment and appears to be curative of most cases. The rarity of this tumour makes it difficult to evaluate its prognosis and natural course.

## Background

Solitary fibrous tumours (SFT) are soft tissue neoplasms of mesenchymal origin first described in 1931 by Klemperer and Rabin [1]. They are typically found in the pleura but are ubiquitously distributed and have been reported to originate from a number of extrapleural sites. Solitary fibrous tumours of the liver (SFTL) are rare, with only 84 reported cases in the English literature (PubMed + Google + publication references) including the present case. Most SFTLs are benign but there have been a handful of reports on malignant cases, some of which have had local recurrences and metastatic spread.

Diagnosis is typically made with histopathological findings and immunohistochemical examination of resected samples. Preoperative investigation of SFTLs can be difficult with non-specific radiological features. Biopsy of radiological liver lesions remains controversial due to the risk of inconclusive results [2, 3] or seeding of the biopsy tract [4]. Given the malignant potential of these tumours, surgical resection is the preferred method of treatment if possible.

This report is only the third described case of its kind in the English literature, a malignant SFTL with extensive local recurrence and metastatic spread 6 years following clear resection margins.

## Main text

### Case presentation

A 61-year-old male was referred to the emergency department by his general practitioner in April 2010 for investigation of loose bowel motions and an episode of black stool. The patient had a history of insulin-dependent type II diabetes mellitus, hypertension, ischaemic heart disease with two previous ischaemic events, obstructive sleep apnoea, depression, schizophrenia and a previous incisional hernia repair.

On examination, he was morbidly obese (BMI 45) and was noted to have marked hepatomegaly. This was not associated with any recent weight loss, haematemesis, jaundice or abdominal pain. The patient denied previous blood transfusions, usage of intravenous drugs and did not drink alcohol. A faecal occult blood test was negative, and the patient’s last colonoscopy 2 years prior was unremarkable.

**Fig. 1:**
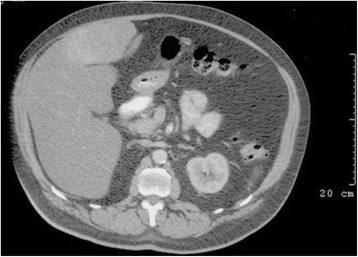
Abdominal CT displaying the pedunculated liver lesion arising from segment IV

**Fig. 2:**
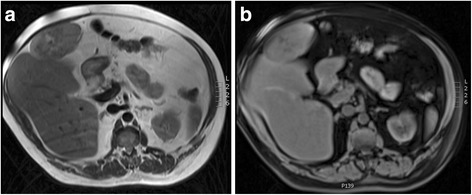
Abdominal MRI displaying the liver lesion. **a** T2WI. **b** T1WI

**Fig. 3:**
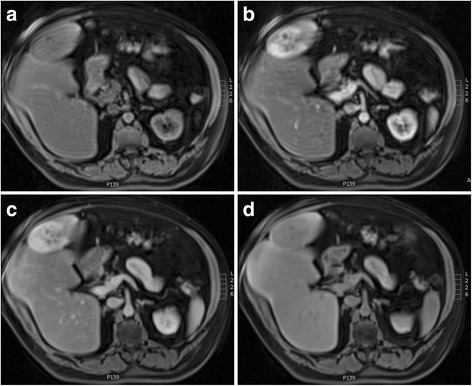
MRI T1WI after contrast. **a** Arterial phase. **b** Portal venous phase. **c** Delayed 2 min. **d** Delayed 10 min

He was referred to our tertiary centre for further management after an ultrasound scan (USS) displayed an ovoid mass of mixed echogenicity arising from the liver, measuring 12 × 9 cm. A computed tomography (CT) scan confirmed a malignant appearing, pedunculated lesion attached to segment IV (Fig. 1). A subsequent magnetic resonance imaging (MRI) confirmed that on T2 weighted imaging (WI), the lesion was isointense to the liver peripherally with central branching hyperintensities (Fig. 2a) which corresponded to the hypointensities seen on T1WI (Fig. 2b). Enhancement of the lesion was noted in arterial phase (Fig. 3a), during portal venous phase (Fig. 3b) and at 2 min (Fig. 3c), with some central areas of non-enhancement. The lesion becomes slightly hypointense on delayed images at 10 (Fig. 3d) and 20 min compared to the surrounding liver.

Laboratory investigations revealed a mildly elevated gamma-glutamyl transpeptidase of 137 IU/L (normal 5–50 IU/L). Hepatitis screen, alpha-fetoprotein, carcinoembryonic antigen and cancer antigen 19–9 were all unremarkable.

The patient underwent a subsegmental resection of the 15 cm segment IVb mass in June 2010. There was severe hepatic steatosis, but no cirrhosis. The patient was discharged postoperative day seven without complications.

Pathology of the resection specimen confirmed SFTL. The specimen displayed a pale tan nodular appearance with a firm and rubbery cut surface. Histological examination revealed fascicles of spindle cells in storiform arrangement with a pushing margin. There was evidence of extracellular collagen deposition, areas of myxoid stroma and branching vessels with hyalinisation. The specimen displayed a high mitotic rate of up to 9 per 10 high-power fields (HPF) with no necrotic or haemorrhagic features. Immunohistochemistry showed positive staining for CD34, CD99 and BCL-2. The tumour was negative for c-Kit, CD31, SMA, desmin, cytokeratins (AE1/AE3, MNF116 and Cam 5.2), EMA and S100. The margins were clear. The non-neoplastic remainder of the liver displayed pericellular fibrosis indicative of steatohepatitis.

**Fig. 4:**
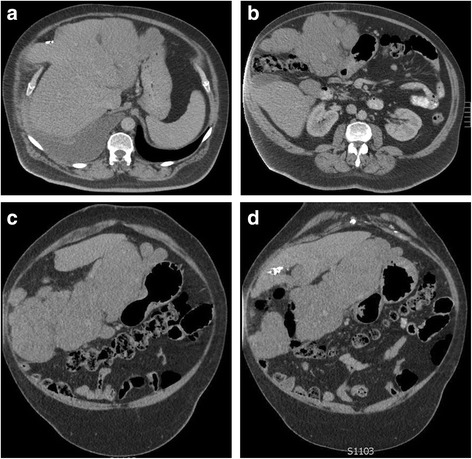
CT abdomen of recurrent disease adjacent to resection site. **a–b** Axial views. **c**–**d** Transverse views

**Fig. 5:**
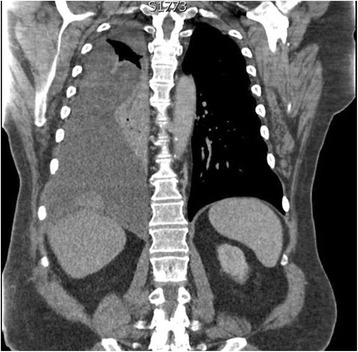
CT chest transverse view displaying right-sided pleural mass associated with significant unilateral pleural effusion

The patient was followed-up regularly every 4 to 6 months with CT scans by the local general practitioner who liaised with the consultant surgeon. There were two episodes of re-admissions for further investigation of recurrent right upper quadrant pain between 2011 and 2013. Multiple MRI scans performed during this period revealed expected postsurgical changes with no tumour recurrence. However, in May 2016, the patient presented to his local emergency department with progressively worsening right upper quadrant pain and increasing dyspnoea with an oxygen demand. CT of his chest, abdomen and pelvis revealed extensive tumour recurrence adjacent to the previous resection site (Fig. 4). In addition, there was a clinically significant right-sided pleural effusion and a pleural mass at the right lung base measuring 3.8 cm (Fig. 5).

Pleurocentesis was performed, draining 1400 ml of serosanguineous fluid. Cytology was negative for malignant cells. The case was discussed extensively in a multi-disciplinary setting, and it was decided given the patient’s two sites of disease and significant perioperative risk that he was not a candidate for radical reoperation. There were also no suitable chemo- or radiotherapeutic therapies available. The patient was subsequently referred to the palliative team for management of his symptoms and discharged back to the community. He was still alive 1 month after discharge.

## Discussion

SFTs are fibroblastic neoplasms first described in 1931 [1] that are of mesenchymal origin and typically arise from the pleura. Initially thought to be of mesothelial origin, they have been historically referred to as benign mesothelioma, localised fibrous mesothelioma and pleural fibromas [5]. Their extrapleural involvement and ubiquitous nature have been well described over the last century with publications documenting primary cases arising from the respiratory tract [6], orbit [7], thyroid [8], adrenal gland [9], spinal cord [10], meninges [11], breasts [12], peritoneum [13], pancreas [14] and soft tissues [15].

SFTs involving the liver are exceptionally rare with only 84 cases reported in the English literature since 1958 (Table 1). The average age of patients is 57.1 (range 16–87) and appears to occur in females more than males (1.4:1). Most SFTLs follow a benign course, however, 17.9% (*n* = 15) of cases displayed malignant histological features.

**Table 1:** Clinical summary of SFTL cases found in English literature

No.	Author	Year	Age	Sex	Lobe	Size (cm)	Hypo	Treatment	IHC	Follow-up
1	Edmondson et al. [57]	1958	16	F	R	23 × 17	N	Resection	n/a	24 months
2			n/a	n/a	R	5 × 5	N	Resection	n/a	n/a
3	Nevius and Friedman [36]	1959	56	M	R	15 × 15	Y	Radiation	n/a	Died after 2 days
4	Ishak et al. [58]	1976	62	M	L	24	N	Resection	n/a	n/a
5			62	F	L	23 × 20 × 13	N	Resection	n/a	Intraoperative death
6	Kim and Damjanov [29]	1983	27	F	L	27 × 23 × 15	N	Resection	n/a	6 months
7	Kottke-Marchant et al. [47]	1989	84	F	L	15 × 9 × 8	N	Resection	V+	29 months
8	Kasano et al. [59]	1991	39	F	L	18 × 10 × 18	N	Resection	n/a	53mo
9	Barnoud et al. [49]	1996	50	M	R	17 × 15 × 11	N	Resection	CD34+, V+	n/a
10	Levine et al. [60]	1997	57	M	L	10 × 18 × 8	N	Resection	CD34+, V+	38 months
11	Guglielmi et al. [27]	1998	61	F	R	20 × 16 × 10	Y	Resection	CD34+, V+	72 months
12	Licesne et al. [61]	1998	69	F	L	n/a	N	Resection	CD34+, V+	12 months
13	Bejarano et al. [16]	1998	49	M	L	17 × 12 × 10	N	Resection	CD34+, V+	15 months
14	Moran et al. [17]	1998	62	F	n/a	23 × 20 × 13	N	Resection	CD34+, V+	n/a
15			34	F	n/a	2 × 0.5	N	Nil	n/a	Incidental (autopsy)
16			57	F	n/a	24 × 19 × 11	N	Resection	CD34+, V+	n/a
17			32	M	n/a	12 × 9 × 7	N	Resection	CD34+, V+	n/a
18			68	F	n/a	17 × 17	N	Resection	CD34+, V+	Died day 2 postop
19			83	F	R	18	Y	Resection	CD34+, V+	Died day 6 postop
20			72	F	L	9	N	Resection	CD34+, V+	12 months
21			62	M	L	24	N	Resection	CD34+, V+	n/a
22			50	F	n/a	3 × 2 × 1.5	N	Resection	CD34+, V+	n/a
23	Fuksbrumer et al. [18]	2000	40	F	R	14–17	N	Resection	CD34+, V+, bcl-2+	n/a
24			71	F	R	14–17	N	Resection	CD34+, V+, bcl-2+	n/a
25			80	M	R	14–17	N	Nil	CD34+, V+, bcl-2+	n/a
26	Yilmaz et al. [30]	2000	25	F	R	32 × 30	N	Resection	V+	6 months
27	Lin et al. [37]	2001	75	M	R	21 × 20 × 18	Y	Resection	CD34+	11 months
28	Gold et al. [62]	2002	n/a	n/a	n/a	n/a	N	n/a	n/a	n/a
29			n/a	n/a	n/a	n/a	N	n/a	n/a	n/a
30	Neeff et al. [63]	2004	63	F	R	30 × 12 × 19	N	Resection	CD34+, V+	6 months
31	Chithriki et al. [32]	2004	76	F	R	20 × 15 × 16	Y	Resection	CD34+, bcl-2+	11 months
32	Vennarecci et al. [19]	2005	65	M	R	30 × 28 × 14	N	Resection	CD34+, V+	30 months
33	Moser et al. [34]	2005	73	F	R	35 × 20 × 15	Y	Resection	CD34+, V+, bcl-2+	n/a
34	Ji et al. [22]	2006	42	F	R	6 × 5 × 5	Y	Resection	CD34+	n/a
35	Lehmann et al. [64]	2006	63	F	R	n/a	N	Resection	CD34+	96 months
36	Nath et al. [44]	2006	61	F	R	21 × 14.5 × 30	N	Resection	CD34+, V+	10 months
37	Terkivatan et al. [28]	2006	74	M	L	24 × 21 × 15	N	Resection	CD34+, CD99+, V+, bcl-2+	12 months
38	Chan et al. [35]	2007	70	M	R	27 × 24 × 12	Y	Resection	CD34+, CD99+, V+, bcl-2+	9 months
39	Obuz et al. [39]	2007	52	M	L	10 × 11 × 12	N	Resection	CD34+, V+	22 months
40	Perini et al. [65]	2007	40	F	L	n/a	N	Resection	CD34+, V+	49 months
41	Weitz et al. [66]	2007	n/a	n/a	n/a	n/a	N	Resection	n/a	n/a
42			n/a	n/a	n/a	n/a	N	Nil	n/a	n/a
43			n/a	n/a	n/a	n/a	N	Nil	n/a	n/a
44	Kandpal et al. [67]	2008	45	F	R	n/a	N	Resection	CD34+	n/a
45	Fama et al. [31]	2008	68	M	R	n/a	Y	Resection	CD34+, V+	25 months
46	Korkolis et al. [3]	2008	82	F	L	18 × 15 × 8	N	Resection	CD34+, V+, bcl-2+, desmin+	21 months
47	Chen et al. [2]	2008	71	M	R	8.7 × 5.5 × 8.5	N	Resection	CD34+, CD99+, bcl-2+	9 months
48	El-Khouli et al. [43]	2008	68	F	L + R	15 × 10.5 × 13	N	TACE	CD34+, V+	n/a
49	Hoshino et al. [21]	2009	30	F	R	6.7 × 4.5 × 4	N	Nil	CD34+, bcl-2+	6 months
50	Novais et al. [50]	2010	34	F	R	25 × 23 × 13	N	Resection	CD34+, V+	24 months
51	Brochard et al. [51]	2010	54	M	R	17	N	Resection	CD34+, V+, desmin+, actin+	72 months
52	Haddad et al. [25]	2010	62	M	L	n/a	N	Resection	CD34+	n/a
53			45	F	R	7.4 × 5.9 × 5.4	N	Resection	CD34+, V+, bcl-2+	n/a
54	Park et al. [68]	2010	51	F	L	n/a	N	Resection	n/a	n/a
55	Peng et al. [52]	2011	24	F	R	30 × 17 × 15	N	Resection	CD34+, V+, bcl-2+	Died at 16 months
56	Sun et al. [69]	2011	59	M	L	9 × 7 × 6	N	Resection	CD34+, CD99+, V+, bcl-2+	24 months
57	Patra et al. [26]	2012	34	F	L	14.5 × 10 × 8	N	Resection	CD34+, V+, bcl-2+	48 months
58	Radunz et al. [33]	2012	85	F	L	n/a	Y	Resection	CD34+, bcl-2+	n/a
59	Belga et al. [70]	2012	66	F	R	n/a	N	Resection	CD34+	30 months
60	Morris et al. [53]	2012	23	F	R	27 × 23.5 × 4	N	Resection	CD34+, V+, bcl-2+	10 months
61	Beyer et al. [45]	2012	46	M	RLig	21 × 7	N	HRT + chemo + resection	CD34+	10 months
62	Soussan et al. [42]	2013	64	M	L	n/a	N	Resection	CD34+, bcl-2+	n/a
63	Liu et al. [71]	2013	42	M	L	1.5 × 1 × 1	N	Resection	CD34+, bcl-2+	n/a
64	Jakob et al. [72]	2013	62	F	L	n/a	N	Resection	CD34+, CD99+, bcl-2+	n/a
65	Debs et al.[73]	2013	65	M	L	n/a	N	Resection	CD34+, CD99+, bcl-2+	12 months
66			87	F	R	14.6 × 12.3 × 17	N	Nil	n/a	10 months
67	Durak et al. [55]	2013	38	F	L	8 × 6 × 2	N	Resection	CD34+, CD99+, SM actin+	n/a
68	Vythianathan and Long [74]	2013	78	M	L	17 × 13	N	Resection	CD34+, CD99+, V+, bcl-2+	n/a
69	Song et al. [75]	2014	49	M	L + R	7.6 × 5 × 4.8	N	Resection	CD34+, V+, bcl-2+	3 months
70	Texeira Jr et al. [76]	2014	68	F	L	7.5 × 6.5 × 5.5	N	Resection	CD34+, V+	28 months
71	Du et al. [56]	2015	55	F	L	11 × 17 × 15	Y	Resection	CD34+, bcl-2+	60 months
72	Beltran [77]	2015	58	M	L	15 × 9 × 6	N	Resection	CD34+, V+	36 months
73	Bejarano et al. [40]	2015	79	F	R	15	N	TACE + resection	CD34+, V+, bcl-2+	31 months
74	Feng et al. [20]	2015	51	M	R	2.3 × 0.3	N	Resection	CD34+, bcl-2+	11 months
75			49	M	L	8.7	N	Resection	CD34+, V+, bcl-2+	17 months
76			51	F	R	8.4	N	Resection + adjuvant chemo	CD34+, V+, bcl-2+	31 months
77			52	F	R	12	N	Resection + MWA	CD34+, V+	37 months
78	Silvanto et al. [24]	2015	65	M	L	18	N	Resection	CD34+, CD99+, bcl-2+	16 months
79	Kueht et al. [23]	2015	40	M	L	4.7 × 4 × 4	N	Resection	CD34+, CD99+, V+, bcl-2+	n/a
80	Maccio et al. [46]	2015	74	F	R	24 × 16	N	Resection	CD34+, V+, bcl-2+, STAT6+	Died at 15 months
81			80	F	R	19 × 15	N	Chemotherapy	CD34+, V+, bcl-2+, STAT6+	Died at 4 months
82			65	M	R	3 × 2	N	Chemotherapy	CD34+, V+, bcl-2+, STAT6+	Died at 5 months
83	Makino et al. [78]	2015	55	M	R	8.6 × 6.3	N	Resection	CD34+, CD99+, bcl-2+	11 months
84	Present case	2016	61	M	R	15 × 11.5 × 7.5	N	Resection	CD34+, CD99+, bcl-2+	74 months

*Hypo* hypoglycaemia, *IHC* immunohistochemistry, *F* female, *M* male, *L* left, *R* right, *N* no, *Y* yes, *n/a* not available, *RLig* round ligament, *TACE* transarterial chemoembolisation, *chemo* chemotherapy, *HRT* hormone replacement therapy, *MWA* microwave ablation

The clinical presentation of SFTL is generally non-specific, ranging from weight loss and fatigue to upper abdominal fullness [16] or discomfort due to the tendency of these tumours to be quite large. In many cases, SFTLs are found incidentally during routine examination [17–22] or on routine imaging while investigating other pathologies [2, 23–25]. Patients may also present with symptoms secondary to compression of visceral or neurovascular structures adjacent to the mass such as dyspepsia [26], postprandial pain/nausea/vomiting [22, 27–29] or jaundice [30]. There is no specific laboratory or tumour marker for SFTL, and serum investigations are generally non-informative. A small percentage of patients (13.1%) however, present with paraneoplastic syndromes such as non-islet cell tumour hypoglycaemia [31] associated with extrinsic production of high-molecular weight insulin-like growth factor II (IGF-II) which spontaneously resolves after resection of the mass [17, 19, 24, 30–37]. There does not appear to be an association between hypoglycaemic presentations and malignant cases (*n* = 1). These IGF-II associated SFTs have also been documented in cases involving the pleura and so are not limited to SFTLs [38].

Preoperative diagnosis is difficult due to non-specific radiological findings. Sonography often reveals a heterogeneous mass which may be either or both hypo- and hyperechogenic with or without calcifications. A contrast-enhanced CT characteristically shows early arterial enhancement with delayed venous washout [3, 22]. Findings on MRI are similar to that of CT scans. In T1WI, the SFTL demonstrates a heterogeneous mass with hypointense signals compared to the normal hepatic parenchyma which is thought to reflect the high content of collagenous tissue [39, 40]. A heterogeneous mass that may be both hypo- and hyperintense is observed in T2WI with some areas described as almost isointense to cerebrospinal fluid [18, 41]. On images post-gadolinium-based contrast injection, SFTLs display progressive heterogeneous enhancement starting in the arterial phase corresponding to the hypervascular areas and persisting into the venous and delayed phases, likely due to the collagen-rich interstitium [42]. There does not appear to be any features on either USS, CT or MRI that differentiates between benign or malignant disease without a tissue diagnosis.

Percutaneous biopsy for tissue diagnosis prior to resection is a much debated topic but is a well-documented approach in the lead up to the resection of SFTLs [3, 16, 18, 27, 28, 31, 40, 43–47]. Given the many risks it poses—including seeding the tumour via the needle tract [4, 48], pain, intrahepatic or subcapsular haematoma and bile leaks [4]—it is doubtful whether preoperative biopsy would change management if the lesion is able to be safely resected [24]. Fuksbrumer et al. [18] describes a case which showed histological changes suggesting low-grade malignant transformation which was not discovered in the initial biopsy while Korkolis et al. [3] reports a case of SFTL whose initial biopsy was indicative of hepatocellular carcinoma. A third report by Chen et al. [2] presents a case in which a biopsy suggested metastatic pancreatic or upper gastrointestinal tract lesion in a patient with a history of colorectal adenocarcinoma prior to resection. Postoperative histological examination indicated SFTL and disproved the preoperative diagnosis.

Diagnosis is limited to histopathological and immunohistochemical investigations. Macroscopic examination of SFTLs appears to be relatively consistent amongst all cases in this literature review. SFTLs range in size, measuring from 0.5 [17] to 35 cm [34]. They tend to be grey-white or tan-yellow in colour and are well-circumscribed, nodular and encapsulated by a smooth glistening capsule, often continuous with the Glisson’s capsule [27, 47, 49]. On the cut surfaces, they are well documented to be firm and difficult to cut with a whorled bulging appearance interspersed with central areas of scarring and radiating bands of fibrous tissue. Some SFTLs may also display features of myxoid degeneration [3, 22, 26, 40, 50], necrosis [20, 27, 28, 35, 42, 51, 52], haemorrhage [20, 51] or cystic cavitation [2, 24, 29, 34, 35, 40, 42, 53].

Microscopically, they are composed of ovoid spindle-shaped cells with little cytoplasm within a characteristic storiform or haphazardly ‘pattern-less pattern’ architecture. These cells are distributed between alternating hypo- and hypercellular areas separated from each other by thick bands of keloid-like collagen bundles and branching of staghorn vessels resembling a haemangiopericytoma-like pattern. Myxoid changes were also commonly observed [17, 26]. Mitoses are rare and generally limited to malignant cases, as is necrosis and cytological atypia. Most cases displayed mitoses <4/10HPF. In 2002, the World Health Organization (WHO) revised their classification of tumours and recognised SFTs as a fibroblastic/myofibroblastic tumour and identified it as a separate entity to haemangiopericytomas. Features identified by WHO to be associated with malignancy include hypercellularity, cytologic atypia, tumour necrosis, infiltrative margins and high mitotic activity (≥4/10 HPF) [54].

There are no specific immunohistochemical profiles for SFTL, however, there are a few markers which are characteristic such as CD34 which has shown strong reactivity in all documented cases as well as CD99, BCL-2 and Vimentin which do not appear as sensitive. Durak et al. [55] reports an interesting case in which there was a strong positivity for CD34 and CD99 but similarly for smooth muscle actin and focal weak positivity for oestrogen and progesterone receptors in the spindle cells which has not been documented before. Few cases report immunoreactivity to desmin [3, 51] (*n* = 2) and actin [51, 55] (*n* = 2). SFTLs are otherwise typically negative for c-Kit (CD117), CD31, cytokeratins, EMA, factor VIII, epithelial membrane antigen and S100.

On literature review, there appears to be sixteen cases documenting malignant SFTLs (Table 2), local recurrence or distant metastases. 17.9% (*n* = 15) of patients were diagnosed with malignant SFTL based on the histology reports. The average age of these patients was 59.6 years with almost equal distribution between males and females (7:8). Of these cases, 26.7% (*n* = 4) were noted to have local recurrence (9 months–6 years) and 53% (*n* = 8) to have distant metastasis (1 month–6 years). This compared similarly to intrapleural SFTs with recurrence rates of 20–67% in malignant tumours [35].

**Table 2:** SFTL cases with malignant features, local recurrence or metastatic disease

No.	Author (year)	Age/sex	Rec/Met	Presentation	Lobe	Size (cm)	Mass (g)	Treatment	Histopathology	Tumour markers	IHC	Follow-up
1	Fuksbrumer et al. (2000) [18]	71/F	Nil	n/a	R	14–17	n/a	Resection (UM)	Dense cellularity, increased nuclear atypia, mitoses 8/10 HPF	n/a	CD34+, bcl-2+, V+	n/a
2	Yilmaz et al. (2000) [30]	25/F	Met	Weakness, fatigue, anorexia, vomiting and progressive jaundice	L + R	32 × 30	4500	Resection (UM)	Cellularity ranged from 20–60%, necrosis, hypervascularity	NAD	V+	Bone metastasis 1 month postsurgery managed with 6 months of chemo (cyclophosphamide, adriamycin)
3	Chan et al. (2007) [35]	70/M	Rec + Met	Hypoglycaemia and progressive jaundice	R	27 × 24 × 12	4400	Failed TACE 6 weeks preoperatively followed by successful resection (UM)	Mildly atypical spindle cells, highly cellular, plemorphia, necrosis, mitoses > 20 HPF	CA-125 145U/ml (normal < 35 U/ml)	CD34+, CD99+, bcl-2+, V+	Asymptomatic widespread bilateral lung metastasis and bi-lobar recurrence at 9 months review
4	Brochard et al. (2010) [51]	54/M	Rec + Met	RUQ pain and weight loss	R	17	n/a	Resection (FM)	Moderately cellular, polymorphic cells, mitoses < 5/10 HPF	GGT 438 IU/ml (normal < 45 U/ml)	CD34+, V+, desmin+, actin+	Local recurrence 6 years postsurgery managed with resection (findings: necrotic, haemorrhagic, highly cellular, moderately atypical nuclei, mitoses >20/10 HPF, CD34, bcl-2. Negative for desmin and actin). Cranial base metastasis managed by embolization and resection. Retroperitoneal and iliac bone metastasis weeks later, patient died 1 month after
5	Peng et al. (2011) [52]	24/F	Met	RUQ discomfort and distention	R	30 × 17 × 15	3750	TACE few days prior to resection (FM).	Highly cellular, pleomorphic, necrosis, mitoses > 10/HPF	CA-125 abnormal	CD34+, bcl-2+, V+	Craniotomy 13 days postsurgery for skull base metastases with large residual lesion. Vertebral metastasis 1 month later managed with 4× PEI and 4 rounds of chemo (adriamycin, ifosfamide, mesna). Tumour relapsed and rapidly enlarged with paralysis on 7 months review, patient died 16 months after initial surgery
6	Belga et al. (2012) [70]	66/F	Nil	Increase in abdominal girth	R	14	n/a	Resection (UM)	Mitoses > 4/10 HPF, necrosis, mild nuclear atypia	NAD	CD34+	30 months
7	Jakob et al. (2013) [72]	62/F	Nil	Upper abdominal pain and weight loss	L	n/a	n/a	Resection (UM)	High cellularity, cytological atypia, necrosis, mitoses 6/10 HPF	NAD	CD34+, CD99+, bcl-2+	n/a
8	Vythianathan and Yong (2013) [74]	78/M	Nil	Epigastric pain	L	17 × 13	n/a	Resection (UM)	Cellular pleomorphism, necrosis, mitoses > 4/10 HPF	n/a	CD34+, CD99+, bcl-2+, V+	n/a
9	Song et al. (2014) [75]	49/M	Nil	Abdominal pain	L + R	7.6 × 5 × 4.8	n/a	Resection (UM)	NAD	n/a	CD34+, bcl-2+, V+	n/a
10	Du et al. (2015) [56]	55/F	Rec	Hypoglycaemia and weight loss	L	15.3 × 15.5 × 15.4	n/a	Resection (UM)	n/a	NAD	CD34+, bcl-2+	Local recurrence 5 years postsurgery, resected
11	Feng et al. (2015) [20]	52/F	Rec	n/a	R	12	n/a	Resection (UM)	Haemorrhage, necrosis	NAD	CD34+	Local recurrence 2 years postsurgery on L lobe managed with PEI. New lesion 6 months after PEI
12	Silvanto et al. (2015) [24]	65/M	Nil	Incidental finding	L	18	n/a	Resection (lesion 1–2 mm from margins)	Myxoid changes, infarction, necrosis mitoses 5–7/10 HPF	NAD	CD34+, CD99+, bcl-2+	16 months
13	Maccio et al. (2015) [46]	74/F	Met	Right abdominal pain and distension	R	24 × 16	n/a	Resection (FM)	Nuclear pleomorphism, cytological atypia, necrosis, haemorrhage, mitoses 9/10 HPF	n/a	CD34+, bcl-2+, V+, STAT6+	Lung, omentum, mesentery and abdominal wall metastasis at 9 months review managed with chemotherapy—patient died 4 months later
14	Maccio et al. (2015) [46]	80/F	Met	Dyspnoea, cough, asthenia, abdominal pain	R	19 × 15	n/a	Palliative chemotherapy	Highly cellular, pleomorphism, necrosis, haemorrhage, mitoses 7/10 HPF	n/a	CD34+, bcl-2+, V+, STAT6+	R lung metastasis managed with palliative chemotherapy—patient died 5 months later
15	Maccio et al. (2015) [46]	65/M	Met	Abdominal discomfort, vomiting and pain	R	3 × 2	n/a	Chemotherapy	Cytological atypia, necrosis, mitoses > 6/10 HPF	n/a	CD34+, bcl-2+, V+, STAT6+	Bilateral lung metastasis managed with chemotherapy, patient died 5 months later
16	Present case (2016)	61/M	Rec + Met	Diarrhoea	R	15 × 11.5 × 7.5	n/a	Resection (FM)	Myxoid changes, mitoses > 9/10 HPF	NAD	CD34+, CD99+, bcl-2+	Extensive local recurrence and pleural metastases 6 years later—palliatively managed. Remains alive 1 month after discharge

*Rec* recurrence, *Met*: metastasis, *IHC* immunohistochemistry, *M* male, *F* female, *L* left, *R* right, *n/a* not available, *UM* unknown margins, *FM* free margins, *HPF* high-power fields, *TACE* transarterial chemoembolization, *RUQ* right upper quadrant, *NAD* no abnormality detected, *PEI* percutaneous ethanol injection, *V* vimentin

Only three cases [35, 51] including the current case are reported to have both local recurrence and metastasis but no significant features to foresee this could be identified in this data. All three were male and their average age was 61.7 years. The size of tumours ranged from 11 to 27 cm and two of the three cases had high rates of mitoses (>9/10 HPF). It is interesting to note that Du et al. [56] reports a case on a 55-year-old female with non-malignant SFTL who represented 5 years after initial surgery with local recurrence and associated hypoglycaemia which resolved spontaneously after resection. The tumour did not display any marked variances when compared to other non-malignant SFTLs.

Surgical resection remains the mainstay of treatment for SFTLs and appears to be the cure for most cases where histopathology returns showing a benign lesion with a clear resection margin of >1 cm. There is very little literature on the use of radio- and chemotherapy, and its efficacy in the long-term is unknown given the scant experience with these approaches. El-Khouli et al. [43] describes a case of SFTL treated with three sessions of transarterial chemoembolisation (TACE) for an inoperable lesion. They describe a favourable outcome on the basis of increased intratumoral necrosis, reduced tumoural enhancement and stabilisation of tumour size based on consecutive MRI scans. Beyer et al. [45] describes a case where the SFTL was initially thought to be a desmoid tumour, and the patient was managed with hormone replacement therapy before imatinib was trialled with no effect. The patient eventually underwent surgical resection with no malignant features evident. Feng et al. [20] presents a case series with one patient undergoing adjuvant chemotherapy (mitomycin) due to extensive tumour infiltration of multiple vascular structures and another patient who was trialled on percutaneous microwave coagulation therapy and percutaneous ethanol injection for local recurrence without success as new lesions were found 6 months later on follow-up. Maccio et al. [46] reports a case series where two patients underwent chemotherapy for SFTL with metastatic spread to the lungs without success as both patients died within 5 months.

Prognosis for SFTLs is unknown and difficult to measure due to the little experience and understanding of the biological nature of the disease. The rarity of this tumour makes it hard to gather enough cases for a study on alternative treatment options, and the absence of long-term follow-up also hinders on the evaluation of patient outcome over long-periods for both benign and malignant cases. The use of adjuvant radio- and/or chemotherapy as well as TACE is scarcely reported and so its efficacy cannot be commented on. It would be valuable to review these patients several years down the track to see how their disease has progressed.

## Conclusion

SFTL is a rare neoplasm that should be considered in the differential diagnosis of patients presenting with vague abdominal symptoms secondary to compression of adjacent structures due to a large hepatic mass. Radiological findings are often non-specific and are unable to differentiate between a benign or malignant mass and percutaneous biopsies are not recommended if the tumour is considered resectable. Complete surgical resection is, thus, the recommended treatment of choice and curative in most cases as the risk for malignant transformation and metastatic spread is not unheard of. Careful long-term follow-up is suggested as prognosis is uncertain for these lesions. This case provides yet another example of the malignant potential of SFTLs to recur locally and metastasise to distant locations.

## Abbreviations

BMI: Body mass index
CT: Computed tomography
HPF: High-power fields
IGF: Insulin-like growth factor
MRI: Magnetic resonance imaging
SFT: Solitary fibrous tumours
SFTL: Solitary fibrous tumours of the liver
TACE: Transarterial chemoembolization
USS: Ultrasound scan
WHO: World Health Organization
WI: Weighted imaging.
